# Leveraging artificial intelligence for analysis of the gut microbiome for dementia diagnosis: a scoping review and discussion

**DOI:** 10.3389/frdem.2026.1865441

**Published:** 2026-06-17

**Authors:** Laya Krishnan, Gaurie Gunasekaran, Tamanna Dhore, Alexa Lauinger, Vijaya Kolachalama, Suguna Pappu

**Affiliations:** 1 Carle Illinois College of Medicine, Urbana, IL, United States; 2 University of Illinois Urbana-Champaign, Urbana, IL, United States; 3 Boston University School of Medicine, Boston, MA, United States; 4 Carle Foundation Hospital, Urbana, IL, United States

**Keywords:** Alzheimer disease, artificial intelligence, brain-gut axis, dementia, gastrointestinal microbiome, machine learning

## Abstract

**Background:**

Dementia, a multifactorial disease with progressive cognitive decline, has been linked to imbalances in the gut-brain axis. Emerging artificial intelligence tools have augmented the identification of several gastrointestinal biomarkers for differential dementia detection and severity, but current literature lacks a comprehensive review.

**Aims:**

This study aims to better quantify the applications of AI in the exploration of the gut microbiome for diagnosis of specific subtypes of dementia.

**Methods:**

Primary articles (*n* = 896) from any point in time through July 2025 were identified from PubMed, Web of Science, Scopus, and ScienceDirect. Title and abstract screenings filtered articles from 896 to 28 for critical appraisal and review for key bacteria, fungi, and metabolites. Methods adhered to the PRISMA extension for Scoping Reviews (PRISMA-ScR) guidelines.

**Results:**

Several studies utilized predictive models including random forests and neural networks to demonstrate alterations in the gut microbiota of Alzheimer’s disease, an increasingly prevalent dementia subtype. These individuals have notably reduced levels of butyrate-producing bacteria, such as *Butyrivibrio*, *Eubacterium*, and *Faecalibacterium*, which contribute to anti-inflammation and gut-barrier maintenance. Increased levels of *Odoribacter splanchnicus* and *Klebsiella pneumoniae,* as well as bacteria from generas *Bacteroides* and *Prevotella,* which help generate short-chain fatty acids, have been implicated in neuroinflammation; *Roseburia inulinivorans* negatively correlates with functional ability. Interestingly, superagers also display unique microbiome profiles that seemingly have protective effects linked to superior cognitive resilience.

**Conclusion:**

Distinct gut microbial compositions are associated with dementia. Furthermore, elucidating gut-brain interactions and their neurodegenerative implications can identify targets for earlier, synergistic diagnostics.

**Systematic review registration:**

https://osf.io/yw2dc/overview.

## 1 Introduction

Dementia is an increasingly prevalent, heterogeneous, multifactorial disease characterized by a progressive loss of memory and cognition. According to the World Health Organization in 2021, there were 57 million people diagnosed with dementia, with about 10 million new cases identified each year (World Health Organization, 2025). Within this overarching disease state, there exist many subclassifications, including fronto-temporal dementia, vascular dementia, and Alzheimer’s disease (AD), each with nuances not only in disease manifestation but also in pathogenesis. A strong limiting factor in the ability to treat dementia is arriving at the initial diagnosis, which can often be delayed due to loosely defined diagnostic criteria and thresholds (Porsteinsson et al., 2021). The emergence of computing as a tool to streamline the diagnostic process is a promising path; furthermore, the ability to objectively discern a currently subjective assessment of dementia offers a more tangible way to define this diagnostic criteria (Chang et al., 2021). In exploring these factors, recent advances in dementia diagnosis have focused on the link between homeostatic imbalances and the gut-brain axis.

The gut microbiome has become a topic of interest in many disease states. It has notably been found to influence metabolic functions and inform the immune system, underscoring its potential for involvement in both diagnostics and treatment (Shreiner et al., 2015). In the last 5 years, artificial intelligence (AI) has exploded the realm of technology’s reach and capabilities, augmenting the power of computing as a tool to sift through exponentially more data than previously possible, and making quantification of vast microbiomes more feasible for biomarker discovery. The development of technology that can decipher data on the terabyte scale has transformed the gut microbiome from a poorly understood black box to a vibrant microsystem with implications for many disease states ranging from cancer to dementia (Allen and Sears, 2019; Verhaar et al., 2022). Importantly, Verhaar’s et al. (2022) paper titled “Gut Microbiota Composition Is Related to AD Pathology” established a link of the gut microbiome with AD using machine learning models, citing a higher odds of amyloid positivity with greater abundances of *Clostridium leptum* and lower abundances of several bacterial species of the *Eubacterium ventriosum*, group. They also demonstrated an association between a lower quantity of bacteria that produce short chain fatty acids with higher odds of positive p-tau status. In 2024, Jiang et al. (2024) used AI to explore the gut microbiome in relation to fatty liver disease, noting 14 specific genes with possible implications and predictive capability in the pathogenesis of nonalcoholic fatty liver disease and AD including the GADD45G and NUPR1. Other studies still have explored how machine learning models can be tweaked with a multitude of integrated inputs (Xue et al., 2024) ranging from demographic data to MRI scans and wellness questionnaires for the purpose of dementia diagnosis.

With respect to literature reviews of the existing landscape, Guo et al. (2023) broadly reviewed AI applications with the biomedical genome from the past 5 years, covering use cases in disease prediction, detection, diagnosis, and treatment. Given that the gut microbiome is more vast in genetic diversity and is capable of inducing changes in the human genome (Allen and Sears, 2019), it becomes an even more intriguing question to utilize AI to see what differences may exist between healthy and disease states. The Guo paper thus provides a basis for Liu et al.’s systematic review paper that surveys the landscape of AI analysis of the gut for liver cirrhosis in particular (Guo et al., 2023; Liu et al., 2018). However, a similar scoping review—through the lens of AI on the gut—has yet to be explored in the context of dementia.

As such, inspired by the research of Verhaar and colleagues, our objective is to address this gap with a scoping review of the ways AI is utilized to explore the gut microbiome for the purpose of identifying bacteria and metabolites as they pertain to dementia diagnosis, classification, and severity.

## 2 Methods

### 2.1 Search strategy and criteria

The PRISMA-ScR (Preferred Reporting Items for Systematic Reviews and Meta-Analyses extension for Scoping Reviews) was followed (Tricco et al., 2018).

To explore the scope of AI evaluation in the gut microbiome for dementia, we completed a search for articles based on the PCC framework: population, concept, and context (Schardt et al., 2007). Texts were included if they met the following criteria: (1) population: humans with a diagnosis of dementia or animal models with induction of dementia, (2) concept: application of AI towards gut microbiome analysis to evaluate or classify the dementia diagnosis, (3) context: all geographical locations, healthcare settings, and ages. The protocol was submitted for registration in the Open Science Framework after study completion for transparency.

### 2.2 Literature inclusion and exclusion criteria

Eligibility criteria included all published primary articles from any point in time, written in English, from the databases PubMed, ScienceDirect, Scopus, and Web of Science based on the search criteria listed in Table 1; search was conducted on July 17, 2025.

**Table 1:** Search criteria used for each database; conducted on July 17, 2025.

Search criteria
*Pubmed:* (Dementia) AND (Machine Learning OR Artificial Intelligence) AND (Gut OR Microbiome) NOT (Review OR Meta-analysis OR Case Report)
*Scopus:* TITLE-ABS-KEY ((dementia) AND (“Machine learning” OR “artificial intelligence”) AND (“Gut” OR “Microbiome”) AND NOT (“review” OR “meta-analysis” OR “case report”))
*ScienceDirect:* dementia AND (gut OR microbiome) AND (machine learning OR artificial intelligence) “Research article” type checkbox selected only.
*Web of Science:* ALL = ((Dementia) AND (Machine Learning OR Artificial Intelligence) AND (Gut OR Microbiome) NOT (Review OR Meta-analysis OR Case Report))

The inclusion criteria were as follows:

- Primary studies that either constructed or utilized an existing AI model based on intestinal microorganisms or metabolites for the diagnosis of dementia.
- Research articles reported in English.

The exclusion criteria:

- Systematic reviews,
- Meta-analyses,
- Case reports,
- Letters,
- Editorials.

### 2.3 Expanded search

In an effort to reduce biases from the aforementioned exclusion criteria, we secondarily conducted a search with queries based on Pubmed’s MESH terms on August 15, 2025 (specifications listed in Supplementary Table S1), and combined the resulting articles with those of the initial search.

### 2.4 Selection process

After the search queries were completed and merged, duplicates were removed. Then, title and abstract screenings based on relevance to the review topic were carried out independently by two study researchers. Only mutually agreed upon articles were kept for the subsequent step; if titles were ambiguous, they were included for abstract screening. No automation tools were used in the process. Original research articles passing these screenings subsequently underwent full-text review for content involving AI methods to assess the gut microbiome in the context of dementia; if so, they met inclusion criteria.

### 2.5 Data extraction, classification, and synthesis

Data charting was performed independently after a thorough reading of each article. Study designs (cross-sectional vs. longitudinal, observational study vs. experimental vs. mixed methods), country of participants, types of dementia studied (people with Alzheimer’s disease, mild cognitive impairment, or dementia broadly), types of samples obtained (fecal samples, CSF, etc.) types of data analyzed (16sRNA, metabolite data), types of machine learning used, and key findings and other characterizing pieces of information were extracted. Because these articles followed various study designs, critical appraisal of these articles was carried out by the same researchers through three tools. The 2018 mixed methods appraisal tool (MMAT) was used for human studies, the Systematic Review Centre for Laboratory Animal Experimentation (SYRCLE) was applied for animal studies, and an unstructured reproducibility checklist was used for computational studies that did not fit either of the two aforementioned types. Because this paper constitutes a scoping review of AI applications and their findings, rather than a systematic evaluation of methodology and potential limitations, appraisal was conducted for completeness, but was not used to determine inclusion (Supplementary Table S2).

Data was synthesized through manually identifying, sorting, and grouping of bacteria and metabolites on an excel spreadsheet with a focus on their impact on increasing or decreasing Dementia and MCI phenotypes. From here, figures and tables were generated for ease of visual interpretation.

## 3 Results

### 3.1 Sources of evidence

**Figure 1:**
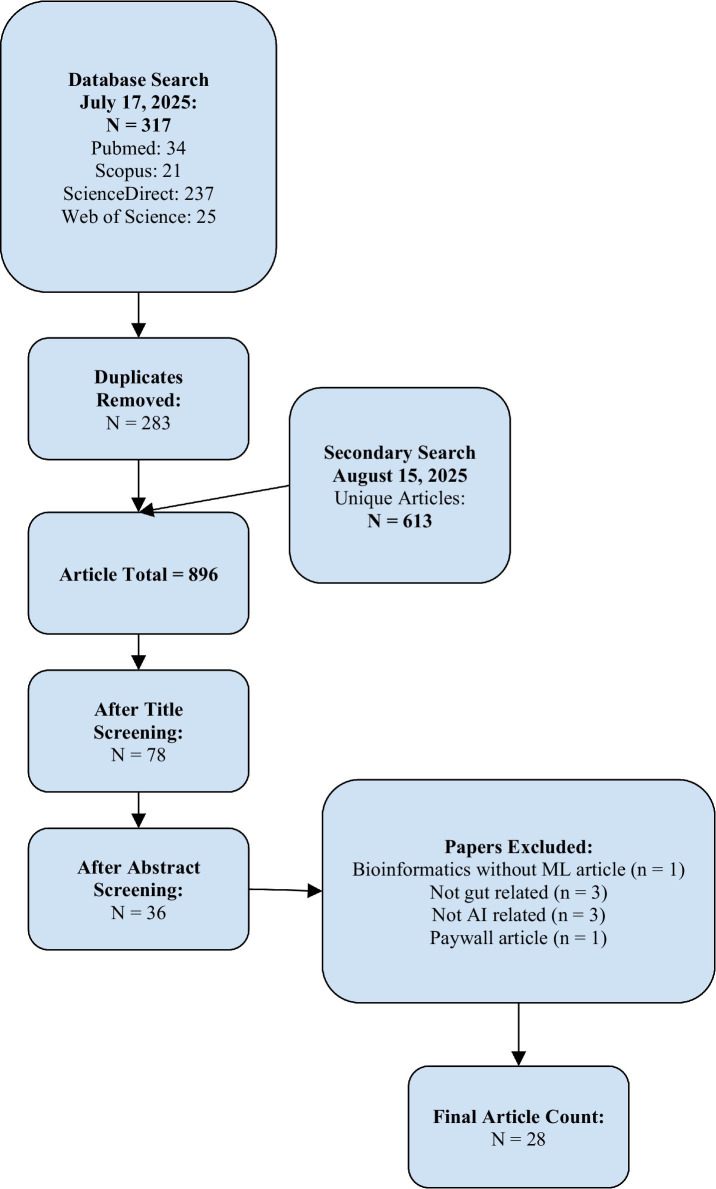
Numbers of sources of evidence screened, assessed for eligibility, and included in the review, with reasons for exclusions at each stage.

**Figure 2:**
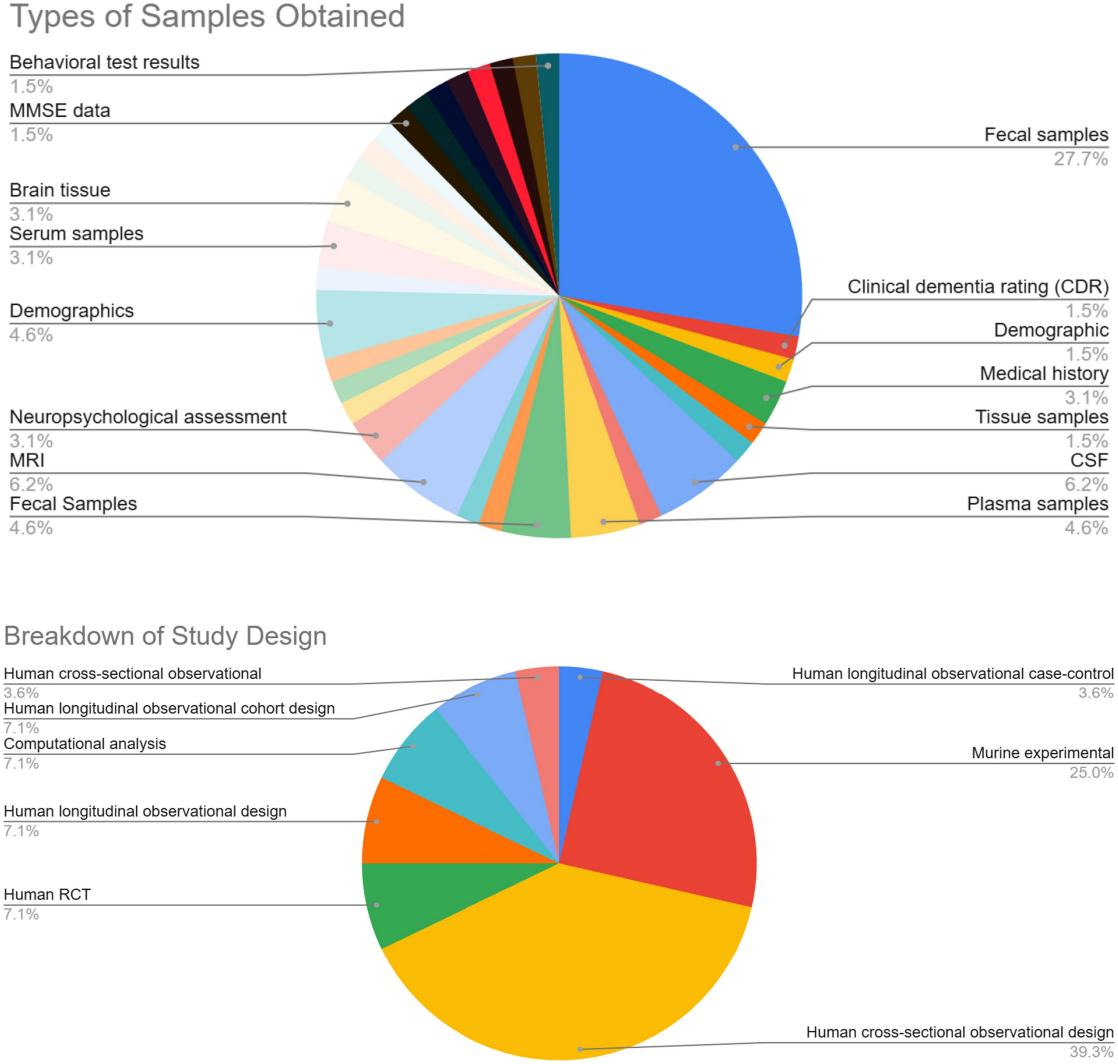
Distribution of types of data samples obtained (top) and study designs (bottom) among all papers reviewed.

The search query was completed, yielding 309 papers, with 283 unique papers after duplicate removal. Including 613 unique papers from the second query resulted in 896. From this original set, title screenings filtered the total to 78 papers, and abstract screenings filtered that total to 36 for full-text review of content. Afterward, articles were removed if content did not include some form of artificial intelligence implementation or involvement of the gut microbiome. This yielded a final paper count of 28 for scoping review and interpretation, all of which were included with critical appraisal with MMAT, SYRCLE, and/or reproducibility checklist scoring. A visual representation of this search method is presented in Figure 1. For all included papers, distributions of types of data samples and study designs are shown in Figure 2, Table 2 demonstrates a comparison of machine learning model performance and other metrics, and Table 3 summarizes microbiome results based on these machine learning methodologies with other study characteristics.

**Table 2:** Machine learning model comparisons of each paper.

**Paper count**	**Paper**	**Machine learning model(s)**	**Training data**	**Testing data**	**Model performance**
1	Haran et al. (2019)	Linear mixed models RF	NS	NS	*Estimated/summarized from figures* Out-of-bag prediction error is < 0.1 for elders without dementia Out-of-bag prediction error is < 0.15 for elders with Alzheimer’s disease
2	Weng et al. (2019)	RF	NS	NS	NS
3	Maj et al. (2019)	PrediXcan VAE SVM RNN Dense Neural Networks CNN	600 samples	208 samples	AUC = 0.862 for DNN-1 AUC = 0.5 for DNN-2 AUC = 0.770 for CNN-1 AUC = 0.5 for CNN-2 AUC = 0.946 for RNN
4	Nagpal et al. (2020)	RF	70%	30%	NS
5	Parikh et al. (2020)	RF	NSNS	NS	*Estimated/summarized from figures* OOB Error % is < 10 for APOE (4 months) OOB Error % is < 30 for Sex (4 months) OOB Error % is < 40 for 5xFAD (4 months) OOB Error % is < 10 for APOE (6 months) OOB Error % is < 40 for Sex (6 months) OOB Error % is < 40 for 5xFA5 (6 months)
6	Rowan-Nash et al. (2020)	DeepARG program	NS	NS	NS
7	Wang et al. (2021)	Multi-model context-sensitive network Network-based prioritization algorithm	NS	NS	NS
8	Huang et al. (2021)	LASSO SVM RF	80%	20%	AUC = 1 for subjects in the P-MCI and AD groups AUC = 0.96 for subjects in P-MCI and S-MCI groups
9	Lu et al. (2022)	RF	NS	NS	AUC = 0.784 for 15 microbial markers
10	Verhaar et al. (2022)	XGBoost	80%	20%	AUC = 0.64 ± 0.10 for the amyloid status AUC = 0.63 ± 0.09 for the p-tau
11	Yıldırım et al. (2022)	RF	NS	NS	AUROC = 0.74 to 1.0
12	Ferreiro et al. (2023)	RF	80%	20%	Accuracy = 0.985 for all biomarkers including Aβ Accuracy = 0.706 clinical covariates + genetic biomarkers Accuracy = 0.674 clinical covariates only
13	Tun et al. (2023)	Artificial intelligence-based KG prediction framework	NS	NS	NS
14	Wang et al. (2023)	LASSO feature selection SVM H2O’s multi-layer feedforward ANN	70%	30%	AUC = 0.873
15	Borsom et al. (2023)	RF	80%	20%	Accuracy = 88.9% for 8 weeks old 3xTg-AD mice Accuracy = 100% for 8 weeks old WT mice
16	Chen et al. (2024)	SNN	NS	NS	Cross-validation score < 1 for cortex Cross-validation score < 3 for serum Cross-validation score < 2 for feces
17	Dunham et al. (2024)	RF	NS	NS	Accuracy = 81% for hAβ-KI HO versus 3xTg-AD HO Accuracy = 91% for hAβ-KI WT versus 3xTg-AD WT
18	Kim et al. (2024)	LightGBM	80%	20%	AUC = 0.832 for the training data set AUC = 0.861 for the test data set
19	Lu et al. (2024)	Supervised and nonsupervised models PLSDA analysis	NS	NS	AUC = 0.7007
20	Wang et al. (2024)	RF GBMEGBoost	NS	NS	AUC: RF (90.29%), GBM (88.80%), XGBoost (89.66%) Average sensitivity: RF (83.29%), XGBoost (85.82%), MLR (77.16%), GBM (75.70%)
21	Laske et al. (2024)	Ensemble-learning model of logistic regressions	NS	NS	AUROC = 0.96 at base line AUROC = 0.96 at 1 year follow-up AUROC = 0.97 at 4 year follow-up
22	Qiu et al. (2024)	Bayesian Ridge CatBoost Elastic Net EGBoost Extra TreesLightGBM GBM RF Ridge Regression K neighbors	70%	30%	R = 0.60 for the Extra Trees Regressor model R = 0.63 for the 10-fold cross-validation Extra Trees model
23	Meng et al. (2024)	SVM RF XGBoost k-nearest neighbors (KNN)	80%	20%	AUC = 1.00 for the baseline training dataset AUC = 0.94 for the day validation cohort AUC = 0.87 for the test set
24	Connell et al. (2024)	RF Naive Bayes AdaBoost	75%	25%	RF AUC = 0.65 AdaBoost AUC = 0.58 Naïve Bayes AUC = 0.63
25	Chen et al. (2025)	LASSO Decision tree RF	NS	NS	RF aMCI: Sensitivity = 0.912, Specificity = 0.947, AUC = 1.00 Decision tree: Sensitivity = 0.958, Specificity = 1.00, and AUC = 0.979.
26	Jia et al. (2025)	RF with Gini impurity	476 samples	1,375 samples	AUC = 0.80
27	Liu et al. (2025)	DNN	70%	30%	AUC = 0.84 ± 0.001 for Total AUC = 0.72 ± 0.002 for ET-E AUC = 0.75 ± 0.001 for ET-F Accuracy = 0.83 ± 0.001 for total Accuracy = 0.65 ± 0.001 for ET-E Accuracy = 0.70 ± 0.001 for ET-F
28	Zhao Y. et al. (2025)	RF	NS	NS	NS

*NS, not specified; RF, random forest; SNN, shallow neural network; DNN, deep neural network; GBM, gradient boosting machine VAE, variational autoencoders.

**Table 3:** Summary characteristics of all reviewed papers.

Paper number	Reference	Country/dataset	Study type	Population	Diagnoses	Data collected	Model used	Results
1	Haran et al. (2019)	USA	Human longitudinal observational case–control	108 patients	AD “Other dementia types”	DNA Cells for flow cytometry MRP2 protein for western blot Shotgun metagenomics	Linear mixed models RF	Lower P-gp expression level induction was seen in stool samples from elders with AD than elders with other types of dementia or the control group
2	Weng et al. (2019)	USA	Murine experimental	NR	AD	16 S rRNA	RF	APOE genotype and sex influenced gut microbiome differences in EFAD miceAPOE4 genotype has a sex-dependent impact on the gut microbiome
3	Maj et al. (2019)	ADNI1- GWAS dataset	Human cross-sectional observational design	808 samples	Dementia CI Early AD Control	16S rRNA tissue-specific cis-eQTL gene expression prediction models SNPS Allelic data	PrediXcan VAE SVM RF RNN Dense Neural Networks CNN	Using a combination of unsupervised and supervised models allows for better performance
4	Nagpal et al. (2020)	USA	Human randomized, double-blind, crossover pilot study design	17 patients	MCI CN	Fungal rRNA	RF	Patients with MCI have higher proportions of families Sclerotiniaceae, Phaffomyceteceae, Trichocomaceae, among others, and lower abundance of Meyerozyma in comparison to their control counterparts
5	Parikh et al. (2020)	USA	Murine experimental	230 mice	AD	16 s rRNA	RF	APOE explained more variance in microbiome composition than sex
6	Rowan-Nash et al. (2020)	USA	Human longitudinal observational design	11 patients	Advanced dementia	16S rRNA from HUMAnN2	DeepARG program	The gut microbiome of patients with advanced dementia was highly variable There were notable fluctuations in the abundance of several species even without antimicrobial treatment No significant differences in pre- and post-levofloxacin samples were noted
7	Wang et al. (2021)	USA	Computational analysis	N/A	AD	Metabolites	Multi-model context-sensitive network Network-based prioritization algorithm	There are links between gut microbial metabolites, microglia, and AD SCFAs are heavily involved in this interaction
8	Huang et al. (2021)	Taiwan	Human cross-sectional observational design	61 patients	Stable MCI, P-MCI, CN	3,749 common features	LASSO SVM RF	A 20-metabolite panel may be useful in predicting the transition mild cognitive impairment to AD Indole-3-propionic acid was associated with AD progression
9	Lu et al. (2022)	China	Human cross-sectional observational design	60 patients	CI	16S rRNA	RF	Cognitively impaired patients had increased levels of Actinobacteria and Proteobacteria A 15 bacterial genera panel may be useful in cognitive impairment prediction
10	Verhaar et al. (2022)	Amsterdam Dementia Cohort of the Netherlands	Human cross-sectional observational design	170 patients	AD MCI SCD	16S rRNA	XGBoost	Lower abundances of SCFA-producing microbes were linked to higher odds of p-tau status and positive amyloid odds
11	Yıldırım et al. (2022)	Turkey	Human cross-sectional observational design	125 patients	AD MCI CN Other neurodegenerative diseases	16S rRNA	RF Latent Dirichlet allocation model Topological data analysis	Taxonomic analysis showed phylum Firmicutes, Bacteroides, Proteobacteria, among others, to be different in MCI and control phenotypes
12	Ferreiro et al. (2023)	USA	Human longitudinal observational design	164 patients	AD	AB plaque from PET Aβ42/Aβ40 in CSF Other tau and genetic biomarkers	RF	Early tau protein and B-amyloid biomarkers were seen in the individuals with early AD in comparison to controls Biomarkers of neurodegeneration were not significantly varied among the groups
13	Tun et al. (2023)	USA	Murine experimental	NR	AD	Proteomics	Artificial intelligence-based knowledge graph prediction framework	Increased levels of integrin β3 and β-galactosidase were associated with increased age and inflammation in AD mice Genetic downregulation of integrin β3 was linked to reduced inflammation
14	Wang et al. (2023)	Alzheimer’s Disease Neuroimaging Initiative (ADNI) Cohort	Human cross-sectional observational design	177 patients	AD CN	Various metabolites including amino acids and microbial metabolites	LASSO SVM H2O’s multi-layer feedforward ANN	GHDCA is consistently correlated with changes in hippocampal structure, biomarkers, and cognitive performance Several important metabolites and biomarkers were associated with AD and control phenotypes
15	Borsom et al. (2023)	USA	Murine experimental	88 mice	AD	16S rRNA	RF	The relative abundance of *Bacteroides* species increased over time in 3xTg-AD mice
16	Chen et al. (2024)	China	Murine experimental	20 mice	AD	16 S rRNA Metabolites	SNN	Dubosiella may protect against AD by producing palmitolic acid, an anti-inflammatory Erysipelatoclostridium is linked to increased AD occurrence
17	Dunham et al. (2024)	USA	Murine experimental	41 mice	AD	Shotgun metagenomic sequencing Metabolomics	RF	There is sex-dependent variance on microbiomes of human amyloid beta knock-in mice AD mice were able to be separated from human amyloid beta knock-in mice based on *Romboutsia ilealis* and *Turicibacter* species
18	Kim et al. (2024)	South Korea	Human cross-sectional observational design	102 patients	SuperagersNormal	16S rRNA	LightGBM	*Alistipes, Leuconostoc*, and others were seen in higher levels in superagers
19	Lu et al. (2024)	China	Human longitudinal observational cohort design	174 patients	AD MCI	Metabolites	Supervised and nonsupervised models	Multiple metabolites had strong correlations with AD biomarkers and APOE4
20	Wang et al. (2024)	China	Human cross-sectional observational design	229 patients	CI CN	16S rRNA	RF GBM EGBoost	Cognitive impairment was associated with lower gut microbiota α-diversity and a less connected network Lower levels of *Megamonas, Blautia, and Veillonella* were seen in cognitive impairment
21	Laske et al. (2024)	Germany	Human longitudinal observational cohort design	49 patients	MCI AD	Shotgun metagenomic sequencing of DNA	Ensemble-learning model of logistic regressions	Age, gender, BMI, and ApoE genotypes were key contributors of MCI phenotypes when determining individuals who are stable versus AD converters
22	Qiu et al. (2024)	USA	Computational analysis	408 human GPCRs 335 gut microbial metabolites	AD	Metabolites	Bayesian Ridge CatBoost Elastic Net EGBoost Extra Trees LightGBM GBM RF K neighbors	Human GPCRs, such as GPR84, were identified as potential AD drug targets Phenethylamine and agmatine were shown to reduce tau hyperphosphorylation in AD neurons
23	Meng et al. (2024)	Turkey	Human phase 2, randomized, double-blinded trial with two arms	87 patients	AD	Untargeted metabolomicsMetagenomics	SVM RF XGBoost KNN	Plasma proteins SKAP1 and NEFL, plasma metabolites homovanillate and glutamade, and *P. clara* were identified to predict AD severity
24	Connell et al. (2024)	United Kingdom	Human cross-sectional observational design	150 patients	CN SCD MCI	16S rRNA	RF Naive Bayes AdaBoost	Many metabolites were significantly altered in preclinical AD. Choline, 5-hydroxyindole acetic acid, and IPA seem to be neuroprotective
25	Chen et al. (2025)	China	Human cross-sectional observational design	74 patients	aMCI CN	NMR metabolomics	LASSO Decision tree RF	Out of 18 identified metabolites, 9 were selected to distinguish between aMCI and NC
26	Jia et al. (2025)	China	Human cross-sectional observational design Small FMT experiment	476 patients	AD MCI NC	Shotgun metagenomic sequencing	RF with Gini impurity	Alterations of gut microbiota can start in very early stages of AD, with over 10% of species showing significant alterations during disease progression
27	Liu et al. (2025)	Europe	Human cross-sectional observational design	317 patients	AD	16S rRNA	DNN	ET-E may be associated with an increased risk of AD while ET-B may be protective
28	Zhao Y. et al. (2025)	China	Murine experimental	27 mice	AD	Brain metabolites 16S rRNA Metabolomics	RF	Eucommiae cortex polysaccharide can alleviate learning and memory impairments in AD Butyric acid impacts glutamine metabolism and brain homeostasis

AD, Alzheimer’s disease; CI, cognitively impaired; CN, cognitively normal; MCI, mild cognitive impairment; SCD, subjective cognitive decline; P-MCI: patients who underwent MCI-to-AD conversion; XGBoost algorithm: Gradient-boosted Tree Models; HUMAnN2: Human microbiome project unified metabolic analysis network 2.

*NS, not specified; RF, random forest; SNN, shallow neural network; DNN, deep neural network; GBM, gradient boosting machine; VAE, variational autoencoders.

### 3.2 Machine learning models used

There is an expanding realm of combinations used in the toolbox of artificial intelligence, which encompasses the range of computation that typically requires human intelligence. This overarching topic can be subdivided into machine learning approaches that learn iteratively and often involve pattern recognition without requiring explicitly defined input. Further subcategorization can include deep learning through neural networks, and predictive modeling that can dynamically forecast outcomes. Among the papers reviewed, the most prominent methods can be categorized with findings as follows. For more complete comprehension, accompanying methods of data preparation and interpretation classically used alongside these machine learning applications have been included in the section below.

#### 3.2.1 Computational methods

│

├── Linear models

│ ├── Logistic regression

│ └── LASSO (least absolute shrinkage and selection operator)

│

├── Tree-based models

│ ├── Random forest (RF)

│ ├── Extra trees regressor

│ ├── XGBoost

│ └── LightGBM

│

├── Ensemble learning approaches

│ ├── Logistic regression + ensemble learning

│ ├── Random forest

│ ├── XGBoost

│ └── LightGBM

│

├── Neural network models

│ ├── Artificial neural network (ANN)

│ ├── Shallow neural network (SNN)

│ ├── Recurrent neural network (RNN)

│ └── Deep neural networks

│ └── Variational autoencoder (VAE)

│

├── Graph-based approaches

│ └── Knowledge graph

│ └── Structured data representation

│

└── Model Interpretation & Explainable AI

└── SHAP (SHapley Additive exPlanations)

└── Applied to models such as LightGBM, RF, XGBoost, etc.

#### 3.2.2 Logistic regression models with ensemble learning

Ensemble learning is a machine learning technique that aggregates multiple models with the intention of maximizing accuracy of prediction. Laske et al. (2024) found maximal accuracy (AUROC of 0.96) from ensemble learning of two functional models from the Kyoto Ortholog and Gene Ontology, and one clinical model (with four parameters of age, gender, body mass index, and Apolipoprotein E genotype).

#### 3.2.3 Tree-based models

Many of these models are based on decision tree-based methods, which are generally capable of both classification (for prediction of discrete, categorical outcomes) and regression (for prediction of continuous numerical values).

*Random forest (RF) model.* RFs are widely successful algorithms that receive their name from their “forest” of decision trees. The algorithm extends the bagging ensemble learning method to handle noise in data and randomly samples a subset of features (with replacement) for value splitting at nodes. These models are frequently used in the context of microbiomic data (Zhang et al., 2022). Weng et al. (2019) used the Boruta algorithm subtype to determine operational taxonomic units significantly able to distinguish samples by Apolipoprotein E (APOE) status and sex compared to randomly generated shadow scores. Haran et al. (2019) similarly used RFs to predict P-glycoprotein expression as a function of the bacterial relative abundances derived from whole-genome sequencing of elderly patients with AD vs. those with other dementia subtypes vs. those who were dementia-free. Furthermore, Nagpal et al. (2020) utilized RFs to explore fungal diversity within the gut microbiome, while Ferreiro et al. (2023) used RFs to pinpoint specific features of the gut microbiome associated with subclinical AD. Chen et al. (2025) used decision tree and RF models to identify a combination of nine optimal metabolic biomarkers that could discern amnestic MCI (AUC = 0.979 and AUC = 1 respectively), with Connell et al. (2024) using them to identify six key metabolites as predictors of early cognitive decline. Jia et al. (2025) similarly employed the model to pinpoint bacteria specific to various stages in the decline of cognitive function in AD. Zhao Y. et al. (2025) used RFs to identify gut genera associated with an increased escape latency in a water maze test—a measure of learning and memory in mice. Borsom’s study also utilized RFs models to demonstrate accurate prediction of 3xTg-AD mice (who have mutations associated with familial AD) 88.9% of the time and WT mice 100% of the time based on gut microbiota (Borsom et al., 2023). Interestingly, Dunham and team found sex-specific distinctions in genotype of the gut metabolomics (Dunham et al., 2024), while Parikh et al. found several bacterial families that were weakly predictive of 5xFAD status, both using RF analysis (Parikh et al., 2020).

*Extra tree regressor.* Extra tree regressors are similar to RF plots in that they create decision trees, however they sample without replacement, and randomly identify split values (as opposed to optimally splitting), allowing for samples unique to a given tree with faster training and greater randomization (Qiu et al., 2024).

*LightGBM and SHAP (SHapley Additive exPlanations) analysis.* LightGBM is also based on decision trees where data is assigned to a bin based on a histogram distribution and tree growth leaf-wise. Furthermore, exclusive features are collapsed together to minimize tree dimensionality, and sampling is nonrandom, instead using Gradient-based One Side Sampling. SHAP analysis helps to interpret LightGBM findings by assigning an impact score for each input feature on a given prediction. Kim and colleagues used this method to identify significant microbiome features for distinguishing superagers (Kim et al., 2024).

#### 3.2.4 Neural network models

*Artificial neural network (ANN).* Artificial neural networks are cornerstones in deep learning, inspired by real animal brain architecture. They use layers of weighted neurons (input, hidden, and output layers), along with backpropagation to mimic synaptic plasticity, in which weights are adjusted based on how often those pathways are traversed, aka the differences between predicted and actual outcomes. Wang and colleagues applied ANNs to assess the utility of various features among many metabolomic biomarkers (Wang et al., 2023).

*Recurrent neural network (RNN).* Recurrent neural networks rely on sequential data inputs to produce sequential predictions, and are thus frequently used in natural language processing. RNNs maintain a consistent weight parameter within each layer of the network (however adjustments are still made in backpropagation), as opposed to CNNs that maintain different weights across each node. Interestingly, Maj and collaborators tested several algorithms, including Logistic Regression (LR), Support Vector Machine (SVM), RF and Deep Learning networks on transcriptomic data (Maj et al., 2019). Building the RNN with the Keras8 and Scikit-learn9 Python libraries on top of TensorFlow yielded a performance accuracy over 90%, allowing them to explore body tissue-specific transcriptomic profiles associated with cognitive decline in AD (Maj et al., 2019).

*Shallow neural network models.* Shallow neural networks, as opposed to deep neural networks, are quite like the name suggests: they have one input layer, one output layer, and a minimal number of hidden layers in between. They are well suited for simpler data, evidenced by Chen and colleagues’ 2024 approach in linking gut microbiota to metabolite levels (Chen et al., 2024).

*Deep neural network models.* Liu et al. (2025) used deep neural networks to narrow down 19 genera of microorganisms found to correlate with AD, and applied SHAP to underscore the two most critical species associated with AD and healthy controls.

Var*iational autoencoder (VAE).* VAEs are neural network architectures that have two main components: an encoder and a decoder. Remarkably, these architectures encode via mapping points within a data set to a latent space rather than a single point, and decode in a similar fashion to map a latent space back to a distribution within the data set. The net effect avoids oversimplification by mapping values to a space that obeys a particular distribution (such as the Gaussian model). Maj et al. (2019) used this algorithm, supplemented with an SVM, to identify APOC2 along with 10 others that are differentially regulated in the putamen of the brain, a region associated with AD.

#### 3.2.5 Linear models

*Least absolute shrinkage and selection operator (LASSO), XGBoost.* LASSO is a regression analysis frequently used in Bayesian statistics that assumes that the majority of coefficients in the linear model are non-zero values. The algorithm then shrinks coefficients towards zero with a penalty term, effectively performing variable selection by eliminating less impactful data features and regularization to prevent overfitting. This method particularly emphasizes the balance between bias and variance to find the optimal lambda value. Huang and collaborators notably used LASSO, SVM, and RF to pinpoint a subset of plasma metabolites with the most relevance in classifying the stable mild cognitive impairment (s-MCI), proceeding to AD with MCI (P-MCI), and AD samples (Huang et al., 2021). Wang and colleagues also effectively distinguished CI and NCI participants using LASSO to identify 16 distinct gut microbiota biomarkers. These biomarkers were then used to construct separate models using Multivariable Logistic Regression (MLR), RF, Gradient Boosting Machine (GBM), and Extreme Gradient Boosting (XGBoost) (Wang et al., 2024). Meng and colleagues (2024) remarkably found that XGBoost outperformed RF and SVM models in identifying select AD gut microbiota among other protein and metabolite features (Meng et al., 2024). They also used k-nearest neighbors (KNNs) to account for limitations in distribution of ADAS-Cog values (representing cognitive impairment) within training datasets.

#### 3.2.6 Graph-based models

*Knowledge graph (Not a model itself, but used as a structured representation of data).* Knowledge graphs are composed of nodes, edges, and labels, where any person, place, or object can define a node and edges define the relationship between them. In the context of the gut microbiome, these structures can be used to better contextualize the complex relationships between various organisms, biomarkers, and genes. Tun and colleagues found use in knowledge graphs to determine rich heterogeneous interactions between the entity and relation embedding to identify novel disease-gene interactions (Tun et al., 2023).

### 3.3 Key findings from AI-driven exploration

Turning our attention now to the derived results of these AI applications, there are certain factors that commonly deviate from normal in the setting of dementia throughout the included studies. These findings can, in part, explain, and themselves be explained by inflammation, a flagship in the discussion of disruptions to homeostasis. Notably, short-chain producing-fatty-acid bacteria, the lesser known mycobiome, and plasma biomarkers (such as tau protein) all appear to play a role in the balance or lack thereof.

#### 3.3.1 Common pathogens increased in AD/MCI phenotypes in comparison to controls

Increases in bacterial abundance from five different phyla—Actinobacteria, Chlamydiae, Firmicutes, Proteobacteria, Spirochaetes, and Bacteroidota—were highly associated with AD and MCI phenotypes across multiple studies (Table 4). Several of the bacteria identified are either established or emerging pathogens, suggesting a potentially critical interplay between the gut microbiota composition, immune system regulation, and neurodegenerative disease.

**Table 4:** Frequently increased and decreased pathogens in AD/MCI patients.

Common pathogens frequently increased in MCI/AD patients	Phylum	Common pathogens frequently decreased in MCI/AD patients	Phylum
*Lactobacillus*	*Firmicutes*	*Collinsella*	*Actinobacteria*
*Helicobacter pylori*	*Proteobacteria*	*Meyerozyma*	*Ascomycota (Fungi)*
*Eggerthella lenta*	*Actinobacteria*	*Escherichia rectale*	*Firmicutes*
*Erysipelatoclostridium*	*Firmicutes*	*Clostridium*	*Firmicutes*
*Escherichia/Shigella*	*Proteobacteria*	*Oscillibacter*	*Firmicutes*
*Streptococcus*	*Firmicutes*	*Fusobacterium*	*Fusobacteriota*
*Chlamydia pneumoniae*	*Chlamydiae*	*Bilophila wadsworthia*	*Proteobacteria*
*Clostridium leptum*	*Firmicutes*	*Enterobacter*	*Proteobacteria*
*Turicibacter*	*Firmicutes*	*Lautropia*	*Proteobacteria*
*K. pneumoniae*	*Proteobacteria*	*Anaeroplasma*	*Tenericutes*
*Sutterella*	*Proteobacteria*		
*Bilophila wadsworthia*	*Proteobacteria*		
*B. burgdorferi*	*Spirochaetes*		

Within the Actinobacterium phylum, *Eggerthella lenta*, an anaerobic emerging pathogen with a well-characterized role in bile acid metabolism, was increased in individuals with dementia-like phenotypes (Haran et al., 2019). Similarly, in the Chlamydiae phylum, *Chlamydia pneumonia*, an obligate intracellular bacterial pathogen implicated in upper respiratory tract infections, was increased in individuals with dementia phenotypes (Angelucci et al., 2019). In the Proteobacteria phylum, elevated levels of *Helicobacter pylori*, a gram-negative bacteria known to cause gastrointestinal ulcers, as well as *Klebsiella pneumonia,* an opportunistic bacteria that colonizes mucosal linings, were noted (Angelucci et al., 2019). Within the Spirochaetes phylum, *Borrelia burgdorferi,* a pathogenic spirochete which causes Lyme disease, was also notably enriched in patients with dementia phenotypes (Angelucci et al., 2019). Within the realm of clinical assessments, Bacteroidota phylum, *Paraprevotella clara* was significantly increased in patients who scored high on the Alzheimer’s Disease Assessment Scale-Cognitive Subscale (ADAS-Cog) in particular (Meng et al., 2024).

These findings are consistent with prior literature that describe increased prevalence of opportunistic pathogens, such as *Ralstonia mannitolilytica*, in individuals diagnosed with AD or other related forms of dementia (Haran et al., 2019). These results along with prior evidence reinforces the need to further evaluate the dysbiosis involving pathogenic or proinflammatory microbes and their contribution to the etiology and progression of dementia.

#### 3.3.2 Common pathogens decreased in AD/MCI phenotypes in comparison to controls

Significant decreases in bacterial prevalence from six different phyla—Actinobacteria, Tenericutes, Firmicutes, Proteobacteria, Meyerozyma, and Fusobacteriota—were consistently associated with AD and MCI phenotypes across multiple studies (Table 4). In literature, several of the bacteria identified in this review are known contributors to pro-inflammatory states (Webb et al., 2016). Thus, their decreased prevalence in dementia or dementia-related phenotypes warrants further investigation into their regulatory or protective roles.

Specifically, within the Actinobacteria phylum, *Collinsella* spp., which has been implicated in several chronic inflammatory diseases such as rheumatoid arthritis and proinflammatory dysbiosis in type 2 diabetes, was found to be decreased in individuals with dementia types in comparison to their healthy controls (Haran et al., 2019). In the Proteobacteria phylum, a decrease in abundance of *Bilophila wadsworthia*, an obligate anaerobe linked to intestinal barrier dysregulation and inflammatory bowel disease, was associated with higher p-tau status among patients (Verhaar et al., 2022). This finding contrasts with other studies reporting increased Bilophila in patients with AD or other dementias, suggesting its dysregulation—increased or decreased—to be a possible indicator of disease pathophysiology and progression (Li et al., 2018). Significantly, *Lautropia* spp., another member of the Proteobacteria phylum known to cause oral inflammation and contribute to the production of proinflammatory cytokines, was decreased among AD Dementia converters in comparison to stable MCI patients, suggesting a potential role in disease progression (Laske et al., 2024).

Further, decreased abundance of several bacteria with known anti-inflammatory properties, such as *Escherichia rectale, Clostridium* spp., and *Oscillibacter* spp.—all belonging to the Firmicutes phyla—were also noted in patients with dementia or related phenotypes (Laske et al., 2024; Chen et al., 2024; Angelucci et al., 2019).

#### 3.3.3 SCFA producing bacteria

Short-chain fatty-acid-producing (SCFA-producing) bacteria have been known to play a vital role in not only gut homeostasis, but also in overall health and wellbeing (Fusco et al., 2023). SCFAs are defined as organic acids that are less than 6 carbons long and typically come from fermentation of indigestible dietary fibers (Verhaar et al., 2022). The most relevant SCFAs include acetate, propionate, and buyrate (C2, C3, and C4, respectively), with acetate consisting of 60% of total SCFAs. SCFAs play several roles in homeostasis, and importantly, regulate the inhibition of histone deacetylase (HDAC). HDAC has been implicated in many neurological disorders, including AD.

Propionate has been shown to be elevated in individuals with AD. Further, Faecalibacterium spp., known for producing SCFAs, was found to be elevated in patients with early AD pathology (Jia et al., 2025). In contrast, pathways that lead to acetate have been shown to have a protective role *in vitro*. Increased levels of *Methanobrevibacter smithii* was shown to be associated with lower concentrations of butyrate. Both acetate and butyrate are protective against Aβ plaque deposition (Ferreiro et al., 2023; Wang et al., 2021). Another study found that SCFA derivatives 5-hydroxyindoleacetate, 4-hydroxyphenylacetate, and 3-hydroxy-2-ethylpropionate were associated with higher cognitive scores based on a standardized mini-mental state exam (MMSE) (Chen et al., 2024).

Many studies have found that SCFA-producing bacteria are less abundant and less diverse in individuals with AD. *Eubacterium ventriosum* spp. (Haran et al., 2019; Chen et al., 2024), *Subdoligranulum* spp., and *Anaerostipes* spp. were the greatest predictors of amyloid status (Verhaar et al., 2022). In addition, *Lachnospiraceae* spp.*, Lachnoclostridium edouardi*, and *Blautia faecis* were the greatest predictors of p-tau levels. *Subdoligranulum* spp.*, Roseburia hominis,* and *Butyricicoccus* spp. were found to be predictors for both amyloid status and p-tau levels. *Faecalibacterium* spp. abundance was also found to be lower in AD (Haran et al., 2019; Yıldırım et al., 2022) as well as *Prevotella* spp. and *Ruminococcus* spp. (Weng et al., 2019).

#### 3.3.4 Mycobiome

While the gut microbiome has become a large topic of interest when studying the gut-brain axis, some researchers note the fungal diversity in the gut, also known as the mycobiome, is often overlooked (Hadrich et al., 2025). While mycobiome studies have improved our knowledge over the past decade, there still remain significant gaps.

One study looked at the effect of gut mycobiome on AD, which they hypothesized to play a role in disease progression. Results indicated that fungal diversity was lower in patients with MCI when compared to cognitively normal patients. However, the difference in overall alpha-diversity was not statistically significant. Still, it was noted that MCI patients tended to have lower levels of the phylum Ascomycota and more so Basidiomycota. More specifically, patients with MCI were found to have lower levels of the family Cladospoiaceae and genus *Meyerozyma*, and higher levels of the genera *Botrytis* and *Cladosporium* along with the family Trichocomaceae, among others (Nagpal et al., 2020).

Specific fungi were also shown to correlate with Aβ-40. For example, in both cognitively normal and MCI patients, Meyerozyma were correlated positively with Aβ-40 and tau while, counterintuitively, Aspergillus was negatively correlated (Nagpal et al., 2020).

#### 3.3.5 Plasma biomarkers

While we did not separately explore the diversity of plasma biomarkers on dementia, several of the reviewed studies tangentially incorporated this variable into their findings, as certain plasma biomarkers have long been known to correlate with AD (Pais et al., 2023). The advantage of having research on these biomarkers is that they are less invasive and costly to acquire. Common biomarkers correlated with AD are Aβ42 (Ferreiro et al., 2023; Yıldırım et al., 2022; Lu et al., 2024), Aβ40 (Ferreiro et al., 2023; Yıldırım et al., 2022), p-tau-181 (Lu et al., 2024), p-tau-217 (Ferreiro et al., 2023; Yıldırım et al., 2022), p-tau-231, t-tau, (Yıldırım et al., 2022), and neurofilament light (Ferreiro et al., 2023; Lu et al., 2024).

The most heavily researched biomarkers are plasma levels of Aβ42 and Aβ40 as well as the Aβ42/Aβ40 ratio. Aβ biomarker levels have been shown to be decreased in AD when compared to cognitively normal patients. This is thought to be because as amyloid plaques form in the brain, there is less available to circulate in the bloodstream. However, it is important to note that Aβ may also be decreased in other amyloid-related pathologies. Meanwhile, tau protein is a much more specific finding. Elevated levels indicate neurodegeneration and have been much more accurate when identifying patients at risk.

#### 3.3.6 Metabolites

Many studies have also investigated the correlation between certain metabolites and AD. One study used Mendelian randomization (MR) to analyze 218 metabolites, out of which they found that 24 were associated with AD. Specifically, 2-hydroxy-4-(methylthio) butanoic acid (HMB), phosphoethanolamine, and glutamine were found to be significant in multiple methods. Additionally, some of these metabolites were associated with bacteria previously known to be linked to AD (e.g., phosphoethanolamine and *Akkermansia muciniphila*). Other bacteria linked to these metabolites include Bacteroides, Bifidobacterium, and Ruminococcus (Qiu et al., 2024). L_alpha_Aminobutyric acid and glycohyodeoxycholic acid have also been found to be correlated with tTau and pTau status, with glycohyodeoxycholic acid additionally playing a role in hippocampal volume (Wang et al., 2023).

Another study looked at over 8,000 metabolite-microglia interactions and found that SCFAs (acetic acid, butyric acid, and propionic acid), ethanol, glutamic acid, dopamine, serotonin, and lactic acid were associated with AD (Wang et al., 2021). Similarly, the following biomarkers were found to be associated with AD: hippuric acid, isocitric acid, tricarboxylic acid (TCA), adipic acid, glucose, and glycohyodeoxycholic acid (Wang et al., 2023). Additionally, it was found that nine metabolites, leucine, valine, glutamate, alpha-glucose, beta-glucose, pyruvate, isobutyrate, phosphocholine, and arginine, as metabolic biomarkers for amnestic mild cognitive impairment (Chen et al., 2025). Several of these metabolites are involved in glucose metabolism, further suggesting a potential correlation between metabolic dysfunction and MCI phenotypes, as a higher fasting plasma glucose concentration was associated with a higher risk of developing AD.

Another article assessed metabolite markers indicated in progressive MCI due to AD versus unprogressive MCI (Lu et al., 2024). Nineteen different metabolites were identified between the progressive and nonprogressive groups, indicating the role certain metabolites may play in disease progression or severity (Lu et al., 2024). A separate investigation looking at plasma metabolites of MCI patients proceeding to AD and stable MCI patients identified indole-3-propionic acid to be a specific metabolite predictor of AD progression (Huang et al., 2021). Indole-3-propionic acid was further determined as one of five serum metabolites, including Indoxyl sulfate, choline, 5-hydroxyindole acetic acid, and kynurenic acid, as altered in preclinic AD in another study (Connell et al., 2024). These recent studies highlight the importance of considering various forms of markers including metabolites in understanding factors associated with dementia and disease progression.

#### 3.3.7 Animal models

Thus far, the aforementioned studies have involved human gut microbiomes. However, animal models have long been utilized to simulate disease states that cannot be as specifically controlled in humans. One such study utilized 4-month male and female E3FAD and E4FAD transgenic mice that overproduce amyloid-*β* 42 and express humanized APOE3+/+ or APOE4+/+ genotypes (Weng et al., 2019). The APOE4 allele, a variant of the common APOE3, has been well characterized as a genetic risk factor for AD, conferring up to a 15 fold increase in risk of developing disease in comparison to the APOE3 individuals (Weng et al., 2019). The gut microbial composition in male E4FAD mice revealed increased relative abundances of bacteria of *Provetella* and *Ruminococcus genera* in comparison to male E3FAD mice (Weng et al., 2019). Similarly, the relative abundance of *Sutterella* was increased in female E4FAD mice in comparison to female E3FAD mice (Weng et al., 2019). A follow up study by the same group reiterated that APOE explained more variance in microbiome composition than sex or 4xFAD. In addition, *Lactobacillaceae* was seen to be increased in APOE4 and reduced in APOE2 mice (Parikh et al., 2020). Meanwhile, *Ruminococcaceae* and *Rikenellaceae* were increased in APOE2 mice, suggesting more efficient resistant starch metabolism and possible microglial benefits (Parikh et al., 2020). A separate investigation noted that there is sex-dependent variance on microbiomes of human amyloid beta knock-in (hAβ-KI) mice compared to wild-type controls (Dunham et al., 2024); female mice had more distinct microbiomes while male mice did not. In addition, female hAβ-KI mice had reduced microbial diversity than wild type controls (Dunham et al., 2024). Early-onset AD mice (3xTg-AD) were able to be separated from human amyloid beta knock-in mice based on *Romboutsia ilealis* and *Turicibacter* spp. (Dunham et al., 2024). These findings highlight the importance of considering sex-specific differences in gut microbial profiling for AD and other dementia types.

Another group interrogating the role of eucommia cortex polysaccharide (EPs) on AD mouse models found that EPs administration alleviated learning and memory deficits (Zhao Z. et al., 2025). EPs administration further reduced β-amyloid deposition in the brains of AD mice and decreased the abundance of *Patescibacteris* and *Actinobacteriota*, which were found to be significantly increased in AD mice compared to controls (Zhao Z. et al., 2025). Similarly, EPs increased the abundance of *Firmicutes, Bacteroidota,* and *Verrucomicrobiota*, which were found to be significantly decreased in AD mice compared to controls (Zhao Z. et al., 2025). These findings suggest the importance of understanding how the gut microbiota can be manipulated to rescue bacterial homeostasis in AD patients. Interestingly, a separate analysis of the microbiota of transgenic female 3xTg-AD mice modeling amyloidosis and tauopathy in comparison to their wild-type (WT) controls noted that the relative abundance of *Bacteroides* spp. increased over time in the 3xTg-AD mice (Borsom et al., 2023). These findings suggest that further research must be conducted to clarify the relationships and roles of these microbial species in regulating AD pathology.

In another study investigating bacterial taxa between AD transgenic mice and WT mice, a significantly increased abundance of the genera *Turicibacter, Dubosiella*, and *Akkermansia* in transgenic mice were noted (Chen et al., 2024). Conversely, significantly lower levels of *Enterobacter, Oscillibacter,* and *Clostridia* genera were determined in the transgenic group in comparison to the WT controls (Chen et al., 2024). The study hypothesizes that specific pathogenic genes such as APP and PSEN1 in AD mice may influence bacterial dysbiosis (Chen et al., 2024), and warrants further exploration into genetic influences within microbial regulation in dementia and related neurodevelopmental diseases.

## 4 Discussion

Understanding the complexities of the gut microbiome and its relationship to dementia is critical in devising treatment options for patients. Despite decades of research efforts, current clinical guidelines rely heavily on medications that attempt to delay disease progression and manage symptoms, further leaning on lifestyle modifications for risk reduction rather than pursuing preemptive disease diagnosis and targeted treatment. This limited array of pharmacotherapeutic interventions is largely due to knowledge gaps in the mechanisms underlying dysbiosis of the gut microbiome and disease etiology. Our review has summarized the identification of several varying categories of bacteria, plasma biomarkers, and the mycobiome that describe the microbiome of the dementia state. Upstream, three main mechanisms contribute to the upregulation and downregulation of these factors.

### 4.1 The peril of frailty and malnutrition on microbiome composition

An important factor that often affects the gut microbial composition is frailty and malnutrition, especially within older community-dwelling adults. Gut dysregulation has been linked to an increased susceptibility for infections; within elderly frail populations, these disruptions can result in clinical decline (Haran and McCormick, 2021).

#### 4.1.1 Human clinical evidence

Other studies have also supported that species diversity decreases with age, with elevated abundances of proinflammatory bacteria present in elderly populations (Hopkins and Macfarlane, 2002). Claesson et al. (2012) suggested that elderly individuals in long-stay care facilities had less diversity of the gut microbial composition in comparison to community dweller controls, which correlated with increased frailty. Interestingly, studies investigating gut microbiota in seniors and centenarians have also noted decreases in bacteria with reported anti-inflammatory properties such as *Faecalibacterium prauznitzii* and relatives in centenarians (Biagi et al., 2010). A pyrosequencing study investigating differences in fecal microbial compositions in 161 individuals of various age groups identified greater Bacteroides spp. and distinct abundance patterns of Clostridium groups in elderly adults in comparison to young adults (Hopkins and Macfarlane, 2002). Similarly, a study investigating frailty in older adults in Korea also associated increased *Bacteriodes fragilis* and *Clostridium hathewayi* with frail adult populations (Lim et al., 2021).

Conversely, good nutrition—including adequate caloric intake, protein, and essential micronutrients—has consistently been associated with decreased rates of frailty. Maintaining sufficient dietary protein in the elderly reduces the risk of sarcopenia, a core component of this metric (Kaur et al., 2019). It has also been shown that overall dietary patterns, such as those who follow Mediterranean diets that are high in fruits, vegetables, and healthy fats, and thus optimize micronutrients such as *α*-carotene, *β*-carotene, and vitamins, are associated with a significantly lower frailty risk (Kobayashi et al., 2013; Boucham et al., 2024). A 3-month study found that prebiotic use was associated with improved frailty, elevated gut probiotic counts, and altered metabolic pathways (Yang et al., 2024). Many such studies have been performed, but there remains limited data on the appropriate dosage of such supplements, highlighting the need for further investigation (Sánchez Y Sánchez de la Barquera et al., 2022).

#### 4.1.2 Murine model evidence

Murine models have also been utilized to determine associations between nutrition, frailty, and AD pathologies. Consistent with findings determined in human studies, increases in *Bacteroides fragilis* within the gut microbiomes have also been linked to cognitive decline and dementia in murine models. In their study, Xia et al. (2023) suggested that recolonization of transgenic mice with live *Bacteroides fragilis* induced AD pathologies. Fascinatingly, treating the mice with metabolites of this species also seemed to provoked AD pathologies.

While studies exploring nutritional diversity and dementia are slowly materializing, these insights on additional microbial-induced variables affecting dysbiosis in elderly populations may be important in understanding disease pathways and progression. Given that the complexity of frailty arises from its domains in malnutrition and inflammation and, while diet may not always be controllable especially for the elderly, pharmacological approaches may represent another area for targeted interventions.

### 4.2 Medications and the gut microbiome

There is also an important consideration to be made in the realm of medication effects on the gut microbiota.

#### 4.2.1 Human clinical evidence

Antipsychotics, which are frequently deployed in the symptomatic presentation of sundowning in AD, were shown to display anti-commensal activity against a pattern of species across several medication subclasses (Maier et al., 2018). Similarly relevant are the effects of antineoplastics, hormones, and compounds targeting the nervous system, all of which were shown to influence the gut microbiome (Patangia et al., 2022).

#### 4.2.2 Murine model evidence

Antibiotics, statins, and fibrates, commonly used medications in the dementia patient population, have implications in the gut microbiome as well (Liu, 2024; Kim et al., 2019). One metagenomic analysis investigating systemic inflammatory states, such as hyperlipidemia, found that fenofibrates correlated with elevated levels of Bacteroides, Bifidobacteria, Allobaculum, and Lactobacilli in the gut microbiota (Liu, 2024). Kim et al. (2019) showed that statins, specifically atorvastatin and rosuvastatin, was associated with an increase in abundance of Bacteroides, Butyricimonas, and Mucispirillum in the gut microbiota.

Several genera of bacteria identified in these studies, such as Bacteroides and Lactobacillus, continue to be extensively studied due to their possible influence on dementia phenotypes (Parikh et al., 2020). Thus, investigating the interconnections between medications, the gut microbiome, and dementia is critical.

### 4.3 Inflammation, cytokine cascades, and dysbiosis

Another potential mechanism of this phenomenon may be characterized by cytokine cascades with resultant increases in pro-inflammatory bacterial colonies.

#### 4.3.1 Human clinical evidence

An observational cohort study analyzing data from 1,337 participants from the Sacramento Area Latino Study of Aging found that IL-6, TNF-a, and CMV IgG were significantly associated with greater errors in the Modified Mini-Mental State Examination (Stebbins et al., 2021). Similarly, a study investigating incident dementia in 732 Korean individuals over the age of 65 found increased serum levels of TNF-α, IL1-α, and IL-1β concentrations to be significantly associated with incidental dementia (Kim et al., 2018).

Further, a Mendelian randomization study identified macrophage migration inhibitory factor (MIF) and basic fibroblast growth factor to be significantly increased in individuals with AD (Ji et al., 2024). However, MIF seemed to be neuroprotective in individuals with Dementia with Lewy Bodies (DLB), suggesting that homeostatic dysregulation of certain cytokines could be associated with the progression or protection of different types of dementia (Ji et al., 2024). MIF, for example, signals through the ERK1/2 which has been shown to activate inflammatory gene expression (Lucas et al., 2022). Dysregulation of such pathways can result in disease. Thus, homeostatic mechanisms and regulation of cytokines and immune mediators within the context of neurodegenerative diseases must be further explored.

Our review has suggested several pro-inflammatory bacteria such as *Helicobacter pylori*, *Escherichia coli, Shigella* spp., and *K. pneumoniae* tend to be increased in individuals with dementia or MCI. *Alistipes, Bacteroides and Prevotella* spp., which are known to be pro-inflammatory and associated with neuroinflammation and neurotransmitter dysregulation, may also be increased in MCI and AD patients as well as patients with severe cognitive impairment (Jia et al., 2025). Additionally, a review article outside of our initial search revealed decreases in anti-inflammatory bacteria were also noted to be present in individuals with dementia or MCI (Angelucci et al., 2019). Many of these bacteria have been shown to affect transcription factors such as NF-kB and Wnt/β-Catenin, which are involved in inflammatory pathways (Ma and Hottiger, 2016). Thus, it makes sense that an increase in pro-inflammatory bacteria or a decrease in anti-inflammatory bacteria would exert similar effects. However, bacteria from the genera *Bifidobacterium and Lactobacillus*, which have been established in exhibiting strong anti-inflammatory properties, were also increased in individuals with dementia or MCI phenotypes amongst various studies (Lu et al., 2022).

#### 4.3.2 Murine model evidence

Murine studies have also suggested a potential interplay between the immune system and cognitive impairments (Gjoneska et al., 2015). Furthermore, it is becoming increasingly evident that systemic inflammation is a contributing factor to cognitive decline (Wakselman et al., 2008).

Similar to human clinical studies, several cytokines, including TNF-a, have been suggestedto exert both neuroprotective effects as well as neurodegenerative effects in murine models (Chertoff et al., 2011). Interestingly, Chertoff et al. (2011) demonstrated that low levels of TNF-a demonstrated protective effects, whereas chronic high levels of TNF-a had a slow, progressive neurodegenerative effect of murine models. Additionally, pro-inflammatory TNF-a has been shown to stimulate neuronal plasticity as well as participate in intracellular calcium buffering (Chadwick et al., 2008).

Furthermore, Weng et al. (2019) showed in their study that *Anaeroplasma* spp., which has been strongly correlated with the secretion of anti-inflammatory intestinal IgA and cytokine TGF-B secretion, was significantly decreased in E3FAD mice compared to E4FAD mice. Thus, investigating the role of gut microbial dysregulation in triggering cytokine cascades which could potentiate neurodegenerative effects is critical in developing treatments for dementia and related diseases.

### 4.4 APOE

APOE is quintessential in the discussion of dementia (Belloy et al., 2023; Bakulski et al., 2021; Nguyen et al., 2020; Preman et al., 2024). While the focus of this review is on lesser known points of exploration, we would be remiss not to emphasize its association with dementia phenotypes. A study utilizing UPLC-MS/MS metabolomics, for example, found X3_Hydroxyisovaleric acid, glycohyodeoxycholic acid, hyodeoxycholic acid, and isolithocholic acid to be associated with APOE4-*ε* in patients (Wang et al., 2023).

## 5 Current gaps in literature

At the present, datasets used for these explorations of the gut microbiome do not encompass intentionally diversified populations, accounting for internal factors such as race, ethnicity, and sex, and external factors including geographical location and socioeconomics. Notably, Rothschild and colleagues found that environmental factors by and large dominate in the formation of the gut microbiome over genetic composition of the host (Rothschild et al., 2018). However, there exist certain well established genomic risk factors for the development of dementia, such as the APOE4 gene described above. While many of these factors are better understood and explored in animal models, studies investigating their role in humans are nascent. Under these notions, epigenetics may help to bridge the gaps between dementia, known genetic predispositions, and microbiome imbalances; together, they may create the perfect storm.

## 6 Limitations and future directions

Our review presents the only article that explores the ways through which AI can be leveraged to assess microbial biomarkers of dementia for earlier detection. However, the articles use heterogeneous methods that inhibit our ability to easily combine these statistics into a meta-analysis; future studies looking into the aggregated results would be insightful. Secondly, several of the studies had a focus on Alzheimer’s disease, or on dementia as a broad category; the emerging nature of this field opens avenues for further analysis and subclassification of dementia for appropriate treatment and surveillance. Finally, the review protocol was registered after study completion.

## 7 Conclusion

Machine learning has shown promising avenues for extensive exploration of the gut microbiome for better detection of dementia and its subtypes. Future research that integrates metabolites, bacteria, fungi, and genetic risk factors of the patient in a longitudinal manner are likely to offer the best breadth of information for AI to process. In overcoming limitations of human subjects research, animal models of AD may serve as an important vehicle to investigate the interplay between gut microbiome disruptions and disease pathology. Furthermore, the optimization of specific features from this breadth using algorithms like LASSO, and using the results to inform machine learning based algorithms may reveal exciting advances in the field of dementia diagnosis, and lend itself to therapeutic targets as a result.

## Data Availability

The original contributions presented in the study are included in the article/Supplementary material, further inquiries can be directed to the corresponding author.

## Supplementary material

The Supplementary material can be found online at: https://www.frontiersin.org/articles/10.3389/frdem.2026.1865441/full#supplementary-material
