# Supplementary material for: Leveraging artificial intelligence for analysis of the gut microbiome for dementia diagnosis: a scoping review and discussion
Source: Front Dement. 2026 Jun 17;5:1865441. doi: 10.3389/frdem.2026.1865441 (PMC13318868; doi:10.3389/frdem.2026.1865441)
Supplement: Supplementary file 1 [file Table_1.docx]

**Table S1**: Search criteria used for each database; conducted on August 15, 2025.

| *Pubmed:* (("Dementia"[Mesh] OR "Frontotemporal Dementia"[Mesh] OR "AIDS Dementia Complex"[Mesh] OR "Dementia, Multi-Infarct"[Mesh] OR "Dementia, Vascular"[Mesh] OR "Alzheimer Disease"[Mesh] OR "Mixed Dementias"[Mesh] OR "Chromosome 3-Linked Frontotemporal Dementia" [Supplementary Concept] OR "Lewy Body Disease"[Mesh] OR "Dementia, familial Danish" [Supplementary Concept] OR "Pick Disease of the Brain"[Mesh] OR "Presenile And Senile Dementia" [Supplementary Concept] OR "Presenile dementia, Kraepelin type" [Supplementary Concept]) OR ( "Alzheimer disease, familial, type 3" [Supplementary Concept] OR "Alzheimer disease type 2" [Supplementary Concept] OR "Alzheimer disease type 4" [Supplementary Concept] OR "Alzheimer disease type 1" [Supplementary Concept] OR "Alzheimer Disease 12" [Supplementary Concept] OR "Alzheimer Disease 5" [Supplementary Concept] OR "Alzheimer Disease 10" [Supplementary Concept] OR "Alzheimer Disease 6" [Supplementary Concept] OR "Alzheimer Disease 7" [Supplementary Concept] OR "Alzheimer Disease 11" [Supplementary Concept] OR "Alzheimer Disease 8" [Supplementary Concept] OR "Alzheimer Disease 9" [Supplementary Concept] OR "Alzheimer Disease 16" [Supplementary Concept] OR "Alzheimer Disease 13" [Supplementary Concept] OR "Alzheimer Disease 14" [Supplementary Concept] OR "Alzheimer Disease 15" [Supplementary Concept] OR "Alzheimer Disease, Early-Onset, With Cerebral Amyloid Angiopathy" [Supplementary Concept] OR "Alzheimer Disease, Familial Early-Onset, With Coexisting Amyloid And Prion Pathology" [Supplementary Concept] OR "Lewy Body Variant of Alzheimer Disease" [Supplementary Concept] OR "Alzheimer's disease without Neurofibrillary tangles" [Supplementary Concept] OR "Alzheimer Disease, Familial, 3, with Spastic Paraparesis and Apraxia" [Supplementary Concept] OR "Alzheimer Disease, Familial, 3, with Spastic Paraparesis and Unusual Plaques" [Supplementary Concept] ) AND ( "Gastrointestinal Microbiome"[Mesh] OR "Brain-Gut Axis"[Mesh] OR "Enteroendocrine Cells"[MESH] OR "Enteric Nervous System"[MESH] OR "Multiomics"[MESH] OR "Dysbiosis"[MESH] OR "Infectious Disease Medicine"[MESH] OR "Metagenome"[MESH] OR "Fecal Microbiota Transplantation"[MESH] OR "Host Microbial Interactions"[MESH] OR "Virome"[MESH] OR "Mycobiome"[MESH] OR "Fatty Acids, Volatile"[Mesh]) AND ("Artificial Intelligence"[MESH] OR "Generative Artificial Intelligence"[MESH] OR "Machine Learning"[Mesh] OR "Machine Learning Algorithms"[Mesh] OR "Transfer Machine Learning"[Mesh] OR "Representation Machine Learning"[Mesh] OR "Reinforcement Machine Learning"[Mesh] OR "Boosting Machine Learning Algorithms"[Mesh] OR "Supervised Machine Learning"[Mesh] OR "Unsupervised Machine Learning"[Mesh] OR "Federated Learning"[Mesh] OR "Extreme Learning Machines"[Mesh] OR "Neural Networks, Computer"[Mesh] OR "Convolutional Neural Networks"[Mesh] OR "Feedforward Neural Networks"[Mesh] OR "Recurrent Neural Networks"[Mesh] OR "Radial Basis Function Networks"[Mesh] OR "Graph Neural Networks"[Mesh] OR "Nerve Net"[Mesh] OR "Deep Learning"[Mesh] OR "Random Forest"[MESH] OR "Decision Trees"[MESH] OR "Ensemble Learning"[MESH])) |
| --- |
| *Scopus:* (TITLE-ABS-KEY("dementia" OR "frontotemporal dementia" OR "AIDS dementia complex" OR "multi-infarct dementia" OR "vascular dementia" OR "Alzheimer disease" OR "mixed dementia" OR "chromosome 3-linked frontotemporal dementia" OR "Lewy body disease" OR "familial Danish dementia" OR "Pick disease of the brain" OR "presenile and senile dementia" OR "presenile dementia, Kraepelin type"  OR "Alzheimer disease, familial, type 3" OR "Alzheimer disease type 2" OR "Alzheimer disease type 4" OR "Alzheimer disease type 1" OR "Alzheimer disease 12" OR "Alzheimer disease 5" OR "Alzheimer disease 10" OR "Alzheimer disease 6" OR "Alzheimer disease 7" OR "Alzheimer disease 11" OR "Alzheimer disease 8" OR "Alzheimer disease 9" OR "Alzheimer disease 16" OR "Alzheimer disease 13" OR "Alzheimer disease 14" OR "Alzheimer disease 15" OR "Alzheimer disease, early-onset, with cerebral amyloid angiopathy" OR "Alzheimer disease, familial early-onset, with coexisting amyloid and prion pathology" OR "Lewy body variant of Alzheimer disease" OR "Alzheimer's disease without neurofibrillary tangles" OR "Alzheimer disease, familial, 3, with spastic paraparesis and apraxia" OR "Alzheimer disease, familial, 3, with spastic paraparesis and unusual plaques"))  AND  (TITLE-ABS-KEY("gastrointestinal microbiome" OR "brain-gut axis" OR "enteroendocrine cells" OR "enteric nervous system" OR "multiomics" OR "dysbiosis" OR "infectious disease medicine" OR "metagenome" OR "fecal microbiota transplantation" OR "host microbial interactions" OR "virome" OR "mycobiome" OR "volatile fatty acids"))  AND  (TITLE-ABS-KEY("artificial intelligence" OR "generative artificial intelligence" OR "machine learning" OR "machine learning algorithms" OR "transfer learning" OR "representation learning" OR "reinforcement learning" OR "boosting algorithms" OR "supervised machine learning" OR "unsupervised machine learning" OR "federated learning" OR "extreme learning machines" OR "neural networks" OR "convolutional neural networks" OR "feedforward neural networks" OR "recurrent neural networks" OR "radial basis function networks" OR "graph neural networks" OR "deep learning" OR "random forest" OR "decision trees" OR "ensemble learning")) |
| *ScienceDirect:* ("dementia" OR "Alzheimer disease" OR "Lewy body disease" OR "Pick disease")AND("gastrointestinal microbiome" OR "gut microbiome" ) AND ("artificial intelligence" OR "machine learning" OR "deep learning")  (“mini reviews” and “review” articles are unselected from side panel) |
| *Web of Science:* (("dementia" OR "frontotemporal dementia" OR "AIDS dementia complex" OR "multi-infarct dementia" OR "vascular dementia" OR "Alzheimer disease" OR "mixed dementia" OR "chromosome 3-linked frontotemporal dementia" OR "Lewy body disease" OR "familial Danish dementia" OR "Pick disease of the brain" OR "presenile and senile dementia" OR "presenile dementia, Kraepelin type" OR "Alzheimer disease, familial, type 3" OR "Alzheimer disease type 2" OR "Alzheimer disease type 4" OR "Alzheimer disease type 1" OR "Alzheimer disease 12" OR "Alzheimer disease 5" OR "Alzheimer disease 10" OR "Alzheimer disease 6" OR "Alzheimer disease 7" OR "Alzheimer disease 11" OR "Alzheimer disease 8" OR "Alzheimer disease 9" OR "Alzheimer disease 16" OR "Alzheimer disease 13" OR "Alzheimer disease 14" OR "Alzheimer disease 15" OR "Alzheimer disease, early-onset, with cerebral amyloid angiopathy" OR "Alzheimer disease, familial early-onset, with coexisting amyloid and prion pathology" OR "Lewy body variant of Alzheimer disease" OR "Alzheimer's disease without neurofibrillary tangles" OR "Alzheimer disease, familial, 3, with spastic paraparesis and apraxia" OR "Alzheimer disease, familial, 3, with spastic paraparesis and unusual plaques") AND ("gastrointestinal microbiome" OR "brain-gut axis" OR "enteroendocrine cells" OR "enteric nervous system" OR "multiomics" OR "dysbiosis" OR "infectious disease medicine" OR "metagenome" OR "fecal microbiota transplantation" OR "host microbial interactions" OR "virome" OR "mycobiome" OR "volatile fatty acids") AND ("artificial intelligence" OR "generative artificial intelligence" OR "machine learning" OR "machine learning algorithms" OR "transfer learning" OR "representation learning" OR "reinforcement learning" OR "boosting algorithms" OR "supervised machine learning" OR "unsupervised machine learning" OR "federated learning" OR "extreme learning machines" OR "neural networks" OR "convolutional neural networks" OR "feedforward neural networks" OR "recurrent neural networks" OR "radial basis function networks" OR "graph neural networks" OR "deep learning" OR "random forest" OR "decision trees" OR "ensemble learning") ) |

**Table S2**: Critical appraisal of all 28 reviewed articles.
Key: 2018 Mixed methods appraisal tool (MMAT; https://www.nccmt.ca/knowledge-repositories/search/232), Systematic Review Centre for Laboratory Animal Experimentation (SYRCLE) risk-of-bias tool.

| **Article** | **Study Design** | **Tool Used** | **Detailed Appraisal (S1, S2 + 5 criteria)** |
| --- | --- | --- | --- |
| **Haran et al., 2019** | Human longitudinal observational case-control | MMAT - Quantitative Non-randomized | S1: Yes.  S2: Yes.  3.1: Partial - case-control sampling  3.2: Yes.  3.3: Partial - missing info limited  3.4: Partial - clinical/diet confounders  3.5: Yes. |
| **Weng et al., 2019** | Murine experimental | SYRCLE | 1. No - No random sequence generation; groups determined genetically (APOE genotype + sex).  2. Yes - Baseline characteristics largely comparable within genetic/sex groups; same age (4 months), same housing and sampling methods.  3. Unclear - Allocation concealment not described.  4. Unclear - Housing conditions and cage distribution not described; random housing not confirmed.  5. Unclear - No mention of blinding of caregivers or experimenters.  6. Unclear - No description of randomization in outcome assessment.  7. Unclear - Blinding of outcome assessors not reported.  8. Unclear - Missing data handling not discussed (exclusion of E2FAD but no statement addressing attrition within analyzed groups).  9. Yes - No evidence of selective reporting; analyses described match stated outcomes.  10. Yes - Other bias present (genetic preselection of groups, exclusion of E2FAD mice, potential cage/sex effects not controlled, cross-sectional sampling only at 4 months). |
| **Maj et al., 2019** | Human cross-sectional observational design | MMAT - Quantitative Non-randomized | S1: Yes.  S2: Yes - ADNI dataset.  3.1: Partial - non-representative cohort  3.2: Yes.  3.3: Partial - missingness not detailed  3.4: Partial - population confounding  3.5: Yes. |
| **Nagpal et al., 2020** | Human Randomized, double-blind, crossover pilot study design | MMAT - RCT | S1: Yes.  S2: Yes.  2.1: Yes.  2.2: Yes - baseline table.  2.3: Yes.  2.4: Partial - ITT unclear.  2.5: Partial - pilot sample. |
| **Parikh et al. 2020** | Murine experimental | SYRCLE  Selection Bias 1. Allocation sequence adequate?  2. Baseline similarity?  3. Allocation concealment?  Performance Bias  4. Random housing?  5. Caregiver blinding?  Detection Bias  6. Random selection for outcome assessment?  7. Outcome assessor blinding?  Attrition Bias  8. Incomplete data handled appropriately?  Reporting Bias  9. Selective reporting avoided?  Other Bias  10. Other problems? | 1. No - convenience sampling; no randomization.  2. Unclear - genotypes defined, but no baseline microbiome equivalence reported.  3. No - genotype/status known; no concealment.  4. No - housed by sex; cage/bedding not controlled.  5. Unclear - no mention of blinding animal handlers.  6. No - fecal samples were samples of convenience.  7. Unclear - no statement on blinding lab personnel.  8. Yes - sample numbers clearly reported; no selective exclusions noted.  9. Yes - all planned analyses reported; raw data deposited.  10. No - acknowledged confounders: non-ideal housing, homozygous APOE, metabolic differences. |
| **Rowan-Nash et al., 2020** | Human longitudinal observational design | MMAT - Quantitative Non-randomized | S1: Yes.  S2: Yes.  3.1: Partial - facility-based sample  3.2: Yes.  3.3: Partial - attrition high (subjects died; missing swabs)  3.4: No - confounders (comorbidities, facility care differences) unaccounted for.  3.5: Yes. |
| **Wang et al., 2021** | Computational analysis | Reproducibility checklist | Methodological Rigor: Strong - Multi-layered network (MGPPN) and random walk prioritization are well-described, but study is purely in silico and largely associative.  Reporting Quality: Partial - Data sources, network construction, and algorithms are detailed, but code and workflow reproducibility are not provided.  Performance Evaluation: Partial - Evaluated with known AD genes, phenotypes, and microbial metabolites, but lacks experimental validation or benchmarking against alternative methods.  Conceptual Soundness: Strong - Aligns with gut-microglia-AD theory; SCFA case study illustrates relevance, though causality and disease specificity are limited. |
| **Huang et al., 2021** | Human cross-sectional observational design | MMAT - Quantitative Non-randomized | S1: Yes.  S2: Yes.  3.1: Partial - small, limited cohort  3.2: Yes.  3.3: Partial - missingness details minimal  3.4: Partial - residual confounders  3.5: Yes. |
| **Lu et al., 2022** | Human cross-sectional observational design | MMAT - Quantitative Non-randomized | S1: Yes.  S2: Yes.  3.1: Partial - population-specific  3.2: Yes.  3.3: Partial - missingness unclear  3.4: Partial - diet/meds unknown  3.5: Yes. |
| **Verhaar et al., 2022** | Human cross-sectional observational design | MMAT - Quantitative Non-randomized | S1: Yes.  S2: Yes.  3.1: Yes - Well-defined cohort.  3.2: Yes - Validated methods.  3.3: Partial - Uncontrolled variables.  3.4: Partial - Residual confounding.  3.5: Yes - Proper analysis. |
| **Yıldırım et al. 2022** | Human cross-sectional observational design | MMAT - Quantitative Non-randomized | S1: Yes  S2: Yes  3.1: Yes - Healthy controls, MCI, and AD patients recruited with clear criteria and exclusions.  3.2: Yes - Validated microbiome and CSF biomarker measurements; appropriate bioinformatics and statistical pipelines.  3.3: Partially - Center variation controlled, but some factors (diet, BMI, stool consistency) not fully controlled.  3.4: Partially - Some confounders controlled; residual confounding from diet, BMI, and stool consistency possible.  3.5: Yes - Data processed and analyzed per protocol with appropriate modeling and validation. |
| **Ferreiro et al. 2023** | Human longitudinal observational design | MMAT - Quantitative Non-randomized | S1: Yes  S2: Yes  3.1: Yes - Adults from longitudinal cohorts; sample representative with clear inclusion/exclusion.  3.2: Yes - Standardized, validated measures for microbiome, biomarkers, and covariates; appropriate analyses.  3.3: Yes - Thorough data collection with imputation for missing values; minor timing gaps between stool and biomarker collection.  3.4: Yes - Controlled for key confounders; some residual confounding possible.  3.5: Yes - Data collection, processing, and predictive modeling conducted as planned. |
| **Tun et al., 2023** | Murine experimental | SYRCLE  Selection Bias 1. Allocation sequence adequate?  2. Baseline similarity?  3. Allocation concealment?  Performance Bias  4. Random housing?  5. Caregiver blinding?  Detection Bias  6. Random selection for outcome assessment?  7. Outcome assessor blinding?  Attrition Bias  8. Incomplete data handled appropriately?  Reporting Bias  9. Selective reporting avoided?  Other Bias  10. Other problems? | 1. No - No random sequence generation; groups determined by genotype.  2. Yes - Baseline characteristics consistent (same background strain, similar housing conditions).  3. Unclear - Allocation concealment not described.  4. No - Animals not randomly housed; genotypes bred separately.  5. Unclear - No statement that caregivers or researchers were blinded.  6. Unclear - Random outcome assessment not reported.  7. Unclear - Blinding of outcome assessors not described.  8. Unclear - No information on incomplete outcome data handling.  9. Yes - No indication of selective outcome reporting; extensive results described.  10. Yes - Other bias present (single background strain, genotype-driven housing, computational analyses dominate over blinded animal outcomes). |
| **Wang et al., 2023** | Human cross-sectional observational design | MMAT - Quantitative Non-randomized | S1: Yes.  S2: Yes.  3.1: Yes - ADNI data; AD, MCI, CN well defined; IRB exemption obtained.  3.2: Yes - Validated cognitive, CSF, MRI, and metabolomic measures.  3.3: Yes - Missing data handled; 70/30 train-test split with cross-validation.  3.4: Yes - Feature selection (LASSO) and predictive modeling (SVM, DL) applied; key confounders included.  3.5: Partial - Moderate sample (n=177), cross-sectional, no longitudinal or alternative ML comparisons. |
| **Borsom et al. 2023** | Murine experimental | SYRCLE  Selection Bias 1. Allocation sequence adequate?  2. Baseline similarity?  3. Allocation concealment?  Performance Bias  4. Random housing?  5. Caregiver blinding?  Detection Bias  6. Random selection for outcome assessment?  7. Outcome assessor blinding?  Attrition Bias  8. Incomplete data handled appropriately?  Reporting Bias  9. Selective reporting avoided?  Other Bias  10. Other problems? | 1. No - No evidence of random sequence generation; groups determined by genotype.  2. Yes - Baseline characteristics similar (same age, same sex, same housing conditions).  3. Unclear - Allocation concealment not described.  4. No - Housing was intentionally not random (genotypes housed separately).  5. Unclear - No explicit blinding of caregivers or investigators reported.  6, Unclear - Random outcome assessment not mentioned.  7. Unclear - No statement that outcome assessors were blinded.  8. Unclear - Handling of missing data not reported.  9. Yes - No signs of selective reporting; outcomes described fully.  10. Yes - Other bias present (cage effects, maternal identity, genotype-segregated housing, single-sex design). |
| **Chen et al. 2024** | Murine experimental | SYRCLE  Selection Bias 1. Allocation sequence adequate?  2. Baseline similarity?  3. Allocation concealment?  Performance Bias  4. Random housing?  5. Caregiver blinding?  Detection Bias  6. Random selection for outcome assessment?  7. Outcome assessor blinding?  Attrition Bias  8. Incomplete data handled appropriately?  Reporting Bias  9. Selective reporting avoided?  Other Bias  10. Other problems? | 1. Yes - Mice segregated by genotype.  2. Yes - Same age, same sex (only males), same housing/diet conditions; groups appear comparable.  3. Unclear - No description of concealment.  4. No - Housing was by genotype (3-4 per cage), intentionally non-random to prevent microbiota mixing.  5. Unclear - No explicit statement of blinding.  6. Unclear - No indication that outcome measurements were randomized.  7. Unclear - Metabolomic and microbiome analyses described, but no mention of assessor blinding.  8. Unclear - No reporting of excluded animals or handling of missing data.  9. Yes - Expected metabolomics/GM analyses were reported comprehensively.  10. Yes - Single-sex design, housing by genotype, and complex multi-omics processing introduce potential confounding. |
| **Dunham et al. 2024** | Murine experimental | SYRCLE  Selection Bias 1. Allocation sequence adequate?  2. Baseline similarity?  3. Allocation concealment?  Performance Bias  4. Random housing?  5. Caregiver blinding?  Detection Bias  6. Random selection for outcome assessment?  7. Outcome assessor blinding?  Attrition Bias  8. Incomplete data handled appropriately?  Reporting Bias  9. Selective reporting avoided?  Other Bias  10. Other problems? | 1. Yes - Animals were grouped by genotype/sex.  2. Yes - Genotypes and control lines were matched and derived from the same origins.  3. No - No mention of concealment of group assignment during allocation.  4. No - Housing was by genotype/sex, which contributed to cage effects.  5. Unclear - Caregivers were likely aware of genotype/group.  6. Unclear - all animals seem included in analyses.  7. Unclear - no mention of blinding for microbiome or metabolomics analysis.  8. Partially - Cage effects and missing metabolite/microbe identification acknowledged; statistical models accounted for cage as a random effect.  9. Yes - Limitations, including unknown species and metabolites, were clearly reported.  10. Yes - Small cohort sizes per cage, high proportion of unassigned reads/metabolites, and potential confounding from environmental/husbandry factors. |
| **Kim et al. 2024** | Human cross-sectional observational design | MMAT - Quantitative Non-randomized | S1: Yes  S2: Yes  3.1: Yes - Community-dwelling adults ≥60 with clear inclusion/exclusion ensuring normal cognition.  3.2: Yes - Standardized cognitive tests and appropriate microbiome collection; only relative abundances used and APOE not included.  3.3: Yes - Complete outcome data for 115 eligible participants; dropouts/refusals documented.  3.4: Partially - Some confounders controlled via eligibility, but no statistical adjustment for others (e.g., APOE ε4).  3.5: Yes - Cognitive testing and microbiome sampling conducted per protocol. |
| **Lu et al. 2024** | Human longitudinal observational cohort design | MMAT - Quantitative Non-randomized | S1: Yes  S2: Yes  3.1: Yes - Clear inclusion/exclusion criteria; representative older adults (≥65).  3.2: Yes - Validated cognitive measures; appropriate biomarker collection.  3.3: Partially - Follow-up completeness unclear; possible missing outcome data.  3.4: Partially - Confounders screened but not statistically adjusted.  3.5: Yes - Exposures and procedures followed as intended. |
| **Wang et al. 2024** | Human  Cross-sectional observational design | MMAT - Quantitative Non-randomized | S1: Yes-clear research question.  S2: Yes-data suitable. 3.1: Partial-community sample but not fully representative. 3.2: Yes-validated MMSE and 16S sequencing.  3.3: Partial-some missing biomarker/lifestyle data.  3.4: No-confounders (diet, lifestyle) not controlled.  3.5: Yes (procedures followed as intended.) |
| **Laske et al. 2024** | Human longitudinal observational cohort design | MMAT - Quantitative Non-randomized | S1: Yes.  S2: Yes.  3.1: Partial - population-specific  3.2: Yes.  3.3: Partial - missingness unclear  3.4: Partial - diet/meds unknown  3.5: Yes. |
| **Qiu et al., 2024** | Computational analysis | Reproducibility checklist | Methodological Rigor: Large-scale multi-omics integration with ML and Mendelian randomization; detailed preprocessing and docking. Limited by GPCR conformational bias.  Reporting Quality: Data and code publicly available; workflow, hyperparameters, and datasets well-described.  Performance Evaluation: Multiple ML models benchmarked against known metabolite-GPCR pairs; docking and MR analyses robust.  Conceptual Soundness: Aligns with gut-metabolite-GPCR-AD theory. Some predictions remain theoretical; in vivo validation and pharmacokinetics not addressed. |
| **Meng et al., 2024** | Human  Phase 2, randomized, double-blinded, placebo-controlled, parallel-group trial with two arms | MMAT - RCT | S1: Yes  S2: Yes  2.1: Yes - Participants were recruited from defined AD cohorts with clear diagnostic criteria for AD severity groups.  2.2: Yes - Exposure and outcome measurements (proteomics, metabolomics, microbiome, imaging, clinical scores) were obtained using validated and standardized procedures.  2.3: Partially - Groups likely differed at baseline (e.g., age, comorbidities), and while covariates were included, full control of all confounders was not explicitly described.  2.4: Yes - Outcome data were largely complete, with multi-omics and clinical assessments available for the analytic sample.  2.5: Yes - Statistical analyses (multi-omics integration, severity stratification, validation cohort) were appropriate and aligned with the study aim. |
| **Connell et al. 2024** | Human cross-sectional observational design | MMAT - Quantitative Non-randomized | S1: Yes  S2: Yes  3.1: Yes - Adults ≥50 from two defined cohorts; clear criteria; groups matched for age, BMI, and sex.  3.2: Yes - Cognitive tests and metabolite/microbiome measures used validated methods; key covariates adjusted.  3.3: Yes - Complete outcome data for all matched participants (n=50 per group).  3.4: Yes - Major confounders (age, BMI, sex, diet, organ function) controlled in design and analysis.  3.5: Yes - Exposures and assessments followed protocol as intended. |
| **Chen et al. 2025** | Human cross-sectional observational design | MMAT - Quantitative Non-randomized | S1 Yes - hypothesis clearly stated.  S2 Yes - high-fidelity NMR and machine learning; adequate analytical detail.  3.1 Yes - Clearly defined inclusion and exclusion criteria, matched for age, sex, and education.  3.2 Yes - groups well matched (age/sex/education).  3.3 Yes - metabolite measurements validated (VIP thresholds; cross-validation).  3.4 Yes - reproducibility and cross-validation clearly described, Matching reduced some confounding.  3.5 Yes - Exposures and analyses were conducted per protocol, including standard metabolomics, cognitive testing, and ML validation. |
| **Jia et al. 2025** | Human cross-sectional observational design  + small FMT experiment | MMAT - Quantitative Non-randomized  and SYRCLE (animal FMT) | S1: Yes - clearly defined multicohort comparison with explicit diagnostic aims.  S2: Yes - validated deep shotgun sequencing; confounders controlled.  3.1: Partial - large sample, but convenient hospital sampling (bias).  3.2: Yes - performed PERMANOVA and covariate correction.  3.3: Yes - validated microbiome + functional annotation.  3.4: Yes - Kraken2/Bracken validated, internal normalization.  3.5: Partial - exclusion criteria not fully reported for dropouts.  SYRCLE (FMT mice): Good randomization reporting, unclear blinding, outcomes appropriate. |
| **Liu et al. 2025** | Human cross-sectional observational design | MMAT - Quantitative Non-randomized | S1: Yes  S2: Yes  3.1: Partially - Participants came from five heterogeneous public datasets, limiting representativeness.  3.2: Yes - Microbiome exposures were processed with standardized pipelines (DADA2, QIIME2, SILVA, PICRUSt2).  3.3: Partially - Outcome completeness is uncertain due to missing metadata (diet, medications, lifestyle).  3.4: Partially - Key confounders were acknowledged but not fully controlled because of incomplete data.  3.5: Yes - Data processing followed established 16S rRNA metagenomic protocols. |
| **Zhao et al. 2025** | Murine experimental | SYRCLE  Selection Bias 1. Allocation sequence adequate?  2. Baseline similarity?  3. Allocation concealment?  Performance Bias  4. Random housing?  5. Caregiver blinding?  Detection Bias  6. Random selection for outcome assessment?  7. Outcome assessor blinding?  Attrition Bias  8. Incomplete data handled appropriately?  Reporting Bias  9. Selective reporting avoided?  Other Bias  10. Other problems? | 1. Yes - Allocation sequence adequately generated.  2. Unclear - Baseline characteristics not fully described.  3. Yes - Allocation concealment reported (researchers/caretakers blinded).  4. Unclear - Random housing not specified.  5. Yes - Caregivers/researchers blinded to group assignments.  6. Unclear - Random outcome assessment not mentioned.  7. Unclear - Assessor blinding for behavioral/microbiome measures not stated.  8. Unclear - Missing data handling not reported.  9. Yes - No evidence of selective outcome reporting.  10. Yes - Other bias present (complex interventions, potential confounding). |
